# Production and Characterization of K562 Cellular Clones Hyper-Expressing the Gene Encoding α-Globin: Preliminary Analysis of Biomarkers Associated with Autophagy

**DOI:** 10.3390/genes14030556

**Published:** 2023-02-23

**Authors:** Matteo Zurlo, Jessica Gasparello, Lucia Carmela Cosenza, Giulia Breveglieri, Chiara Papi, Cristina Zuccato, Roberto Gambari, Alessia Finotti

**Affiliations:** 1 Department of Life Sciences and Biotechnology, Section of Biochemistry and Molecular Biology, University of Ferrara, 44121 Ferrara, Italy; 2 Center ‘Chiara Gemmo and Elio Zago’ for the Research on Thalassemia, University of Ferrara, 44121 Ferrara, Italy

**Keywords:** β-thalassemia, α-globin gene, autophagy, ULK-1, apoptosis

## Abstract

One of the most relevant pathophysiological hallmarks of β-thalassemia is the accumulation of toxic α-globin chains inside erythroid cells, which is responsible for their premature death (hemolysis). In this context, the availability of an experimental model system mimicking the excess in α-globin chain production is still lacking. The objective of the present study was to produce and characterize K562 cellular clones forced to produce high amounts of α-globin, in order to develop an experimental model system suitable for studies aimed at the reduction of the accumulation of toxic α-globin aggregates. In the present study, we produced and characterized K562 cellular clones that, unlike the original K562 cell line, stably produced high levels of α-globin protein. As expected, the obtained clones had a tendency to undergo apoptosis that was proportional to the accumulation of α-globin, confirming the pivotal role of α-globin accumulation in damaging erythroid cells. Interestingly, the obtained clones seemed to trigger autophagy spontaneously, probably to overcome the accumulation/toxicity of the α-globin. We propose this new model system for the screening of pharmacological agents able to activate the full program of autophagy to reduce α-globin accumulation, but the model may be also suitable for new therapeutical approaches targeted at the reduction of the expression of the α-globin gene.

## 1. Introduction

β-thalassemias are a group of hereditary hematological pathologies caused by more than 300 mutations of the adult β-globin gene, leading to low or absent production of adult hemoglobin (HbA) [1,2,3,4]. β-thalassemias are among the most impactful diseases in developing countries, mainly due to the lack of genetic counselling and prenatal diagnosis, which is responsible for the very high frequency of the disease in the population [1,2].

The pathophysiology of β-thalassemia is also caused by an imbalance between α- and β-globin chains, with an excess of free α-globin chains, causing ineffective erythropoiesis and hemolysis [5,6]. Several studies sustain the concept that when β-thalassemia coexists with alterations of the number of α globin genes, the clinical phenotype of thalassemia could change from severe anemia, in the case of a triplication of the α globin genes, (αα/ααα) to mild anemia in the case of an α-globin gene deletion [5,6,7,8]. Therefore, the reduction of free α-globin chains has a clear clinical impact, as suggested by a large number of studies [9,10,11,12] demonstrating that when α-thalassemia is co-inherited with β-thalassemia, the excess free α-globin chains is reduced significantly, ameliorating the clinical severity. Furthermore, Lechauve et al. [13] demonstrated an important role of the autophagy-activating kinase ULK-1 (Unc-51-like kinase 1) in promoting the controlled clearance of free α-globin chains. Using a β-thalassemic mouse model, they found that the loss of the ULK1 gene reduces autophagy, exacerbating disease phenotypes and leading to a lack in the clearance of α-globin in red blood cell precursors; these data confirm that α-globin accumulation is one of the most serious aspects in the pathophysiology of β-thalassemia. Interestingly, Lechauve et al. demonstrated that rapamycin could induce ULK1-dependent autophagy and contribute to α-globin clearance in erythroid cells [13].

With the objective of decreasing α-globin gene expression, Mettananda et al. [14] demonstrated the use of CRISPR/Cas9 genome editing to mimic a natural mutation, which deletes the MCS-R2 α-globin enhancer and causes α-thalassemia. When edited CD34+ cells from β-thalassemia patients were differentiated as erythroid cells, they observed the expected reduction in α-globin expression and a correction of the globin chain imbalance, suggesting that this CRISPR-Cas9-based approach might be of clinical relevance [14]. A second study on this very important issue was published by Pavani et al. [15], demonstrating the correction of the pathological phenotype of β-thalassemia by the CRISPR/Cas9 editing of the α-globin locus in human hematopoietic stem cells.

Another interesting approach to partially solve α-globin/β-globin chain imbalance is to target α-globin mRNA with an antisense oligonucleotide, and this strategy was demonstrated to be effective in murine [16] and human [17] isolated erythroid β-thalassemia cells.

In this context, the availability of an experimental model system that mimics the excess in α-globin chain production and that is prone to autophagy and apoptosis could be of great interest in the development of protocols aimed at reducing free α-globin chains and activating autophagy. Unfortunately, one of the most commonly used cellular systems, the erythroleukemia K562 cell line [18,19,20], exhibits an α-thalassemic-like phenotype characterized by a low transcription of α-globin genes and an absent production of α-globin protein [21]. The K562 cell line was selected for this study as well characterized with respect to its response to several fetal hemoglobin inducers, including rapamycin (sirolimus), which was recently proposed as a potent modulator of autophagy in erythroid cells, with an associated decrease in free α-globin chains [13].

The objective of the present study is to produce and characterize K562 cellular clones forced to produce high amounts of α-globin, in order to develop an experimental system suitable for studies aimed at the induction of the full program of autophagy, previously demonstrated to be of great relevance for decreasing the excess free α-globin chains in erythroid cells of β-thalassemia patients, thereby ameliorating their clinical status.

## 2. Materials and Methods

### 2.1. Cell Culture of Human Erythroleukemic K562 Cells (K562 Wild Type) and Obtained Clones

K562 WT cells were isolated and characterized by Lozzio CB and Lozzio BB, from a patient with chronic myelogenous leukemia (CML) in a terminal blast crisis [22]. K562 WT cells were cultured in a humidified atmosphere of 5% CO_2_, in a RPMI-1640 medium (Euroclone, Pero, Italy, cat. n. ECB2000) supplemented with 10% fetal bovine serum (FBS; Biowest, Nuaillé, France), 50 units/mL penicillin and 50 μg/mL streptomycin (Euroclone, Pero, Italy, cat. n. ECB3001), while the obtained K562 clones were maintained in the same medium supplemented with 0.6 mg/mL of Hygromycin B (MedChemExpress, Monmouth Junction, NJ, USA, cat. n. HY-B0490) as a selection antibiotic (this concentration was selected experimentally by plotting a kill curve as shown in Supplementary Figure S2).

### 2.2. Production of K562 Clones Hyper-Expressing α-Globin

For the production of K562 cell clones with integrated copies of a α-globin-expressing vector, we transfected K562 cells with the pcDNA3.1/Hygro(+)/α-globin vector and then selected positive cells by limiting dilutions in the presence of Hygromycin B selection antibiotic. Detailed information about the cloning process is provided in Supplementary methods.

### 2.3. Cell Proliferation Analysis and Apoptosis Profile

The cell number/mL was monitored using a model Z2 Coulter counter (Coulter Electronics, Hialeah, FL, USA), while the apoptosis profile was measured by employing the Guava^®^ Muse^®^ Cell Analyzer instrument (Luminex Corp., Austin, TX, USA) and its related kit according to the instructions supplied by the manufacturer. Briefly, a 100 μL cell suspension was incubated with 100 μL of the Muse^®^ Annexin V & Dead Cell reagent (cat. n. MCH100105) at room temperature and protected from light for 20 min. The samples were then analyzed using Guava^®^ Muse^®^ Cell Analyzer and data were acquired by utilizing Annexin V and Dead Cell Software Module (Luminex Corp., Austin, TX, USA), as previously reported [23].

### 2.4. RNA Extraction

Total RNA extraction was performed with the classical method of phenol-chloroform extraction and by using TRI Reagent (Sigma-Aldrich, St. Louis, MO, USA, cat. n. 93289), as reported elsewhere [24]. The extracted RNA was stocked at −80 °C in 75% ethanol until the time of the analyses; before proceeding with quantification and the reverse transcription reaction, the obtained RNA samples were centrifuged, the ethanol was removed by aspiration and finally, the RNA pellets were resuspended in nuclease-free water.

### 2.5. RT-qPCR Analysis

The reverse transcription reaction of the RNA was performed using the TaqMan Reverse Transcription Reagents kit (Applied Biosystems, Foster city, CA, USA, cat. n. N8080234) following the manufacturer’s protocol; 500 ng of total RNA was used as the substrate of the reaction to produce single-chain cDNA. At the end of the reaction, the cDNA samples were rapidly centrifuged and stored at −80 °C until their use.

A quantitative real-time multiplex PCR assay was carried out using two different reaction mixtures, the first one containing α-globin or ULK1 probes and primers, and the second one containing GAPDH, RPL13A, β-actin probes and primers (employed as reference genes to normalize data). The sequences of primers and probes (IDT, Tema Research, Castenaso, Italy) used in the multiplex quantitative PCR reactions are shown in Table 1.

Every polymerase chain reaction was performed in double, and in all the Real Time quantitative-PCRs performed, we used no template controls and several scalar concentrations of non-treated samples to ensure the quality of the analysis.

Each reaction mixture contained 1× TaKaRa Ex Taq^®^ DNA Polymerase (Takara Bio Inc., Shiga, Japan, cat. n. RR39WR), 500 nM forward and reverse primers and the 250 nM probes (Integrated DNA Technologies, Castenaso, Italy), as previously reported [25]. The assays were carried out using CFX96 Touch Real-Time PCR System (Bio-Rad, Hercules, CA, USA). The amplification program used consisted of an initial denaturation step at 95 °C for 1 min followed by 50 cycles consisting of two phases: 95 °C for 15 s (denaturation) and 60 °C for 45 s (annealing and extension).

The data were analyzed by employing the CFX manager software (Bio-Rad, Hercules, CA, USA); to compare the gene expression of each template amplified, the 2^−ΔΔCt^ method was used.

### 2.6. Western Blotting Analysis

For extract preparation, the cells were lysed in an ice-cold RIPA lysis buffer (Thermo Fisher, Waltham, MA, USA, cat. n. 89900). Briefly, K562 cells (1 × 10^6^ cells) were collected and washed twice with cold PBS; the cellular pellets were then resuspended with 80 μL of a cold RIPA buffer, incubated on ice for 20 min (vortexing every 3 min) and finally centrifuged at 14,000× *g* for 20 min at 4 °C; the supernatants were collected and immediately frozen at −80 °C; the protein concentration was determined using Pierce™ BCA Protein Assay Kit (Thermo Fisher, Waltham, MA, USA, cat. n. 23225) according to the manufacturer’s instructions.

Twenty μg of cytoplasmic extracts were denatured for 5 min at 98 °C in a 1× SDS sample buffer (Cell Signalling Technology, Danvers, MA, USA, cat. n. 7722) and loaded on hand casted SDS-PAGE 14% polyacrylamide gel (10 cm × 8 cm) in Tris-glycine Buffer. The Spectra pre-stained multicolor protein ladder (Thermo Fisher, Waltham, MA, USA, cat. n. 26634) was used as a standard to determine the molecular weight (size range 10–260 kDa) together with Bio-Rad Precision Plus Protein WesternC Standard (Bio-Rad, Hercules, CA, USA, cat. n. 1610376) or the NEB pre-stained protein marker (New England Biolabs, Ipswich, MA, USA, cat. n. P7708S). The electrotransfer to a 0.2 μm pore- sized nitrocellulose membrane (Thermo Fisher, Waltham, MA, USA, cat. n. 77012) was performed overnight at 360 mA and at 4 °C in a standard Tris-Glycine transfer buffer. The membranes were stained with Ponceau S Solution (Sigma-Aldrich, St. Louis, MO, USA, cat. n. A40000279) to verify the transfer, washed with TBS (10 mM Tris–HCl pH 7.4, 150 mM NaCl) for 10 min at room temperature and incubated in a blocking buffer (5% milk in TBST) for 1 h at room temperature. The membranes were washed three times with TBST (TBS, 0.1% Tween-20) and incubated with the primary antibody in 5% BSA in TBST while being gently shaken overnight at 4 °C. The next day, the membranes were washed three times with TBST and incubated in a blocking buffer while being gently shaken for 2 h at room temperature, with an appropriate HRP-conjugated secondary antibody. Finally, after three washes with TBST, each membrane was incubated with a LumiGLO^®^ detection mix according to the manufacturer’s instructions (Cell Signaling Technology, Danvers, MA, USA, cat. n. 7003S) and imaged directly with the ChemiDoc MP imager (Bio-Rad, Hercules, CA, USA). In order to re-probe the membranes, they were stripped using Restore™ Western Blot Stripping Buffer (Thermo Fisher, Waltham, MA, USA, cat. n. 21059) and incubated at 30 min at 37–42 °C with moderate agitation. Blot images were acquired and analyzed using Bio-Rad Image Lab Software (Bio-Rad, Hercules, CA, USA). The list of employed primary and secondary antibodies is reported in Table 2.

### 2.7. FACS Analysis

For autophagy detection by flow cytometry in K562 cells, Cyto-ID autophagy detection kit 2.0 (Enzo LifeSciences, Farmingdale, NY, USA, cat. n. ENZ-KIT175) was employed. For this analysis, one million cells were washed in PBS before proceeding staining.

The cells were stained with 250 μL of 1:1000 Cyto-ID diluted in PBS and incubated 30 min inside the incubator. Then, the cells were washed in PBS and stained with LIVE/Dead™ Fixable Aqua—Dead Cell Stain Kit (Thermo Fisher, Waltham, MA, USA, cat. n. L34957) and incubated for 10 min in the dark. After an additional wash step, the cells were resuspended in 200 μL of PBS and analyzed by flow cytometry using the BD FACSCanto II cell analyzer (Becton Dickinson, Franklin Lakes, NJ, USA). The control cells (not treated), as well as the treated cells, were additionally co-treated with 40 μM chloroquine for 24 h to better detect the shift in autophagic flux [26].

### 2.8. Genomic DNA Isolation from Whole Blood or Cultured Cells

Genomic DNA isolation from cultured cells was performed with the GRS Genomic DNA kit (GRiSP, Porto, Portugal, cat. n. GK06.0100), as reported elsewhere [27]. The extraction of genomic DNA was performed on one million cells for the checking of cloned cells, or 300 μL of fresh blood to proceed with α-globin gene isolation.

### 2.9. Agarose Gel Electrophoresis

Agarose gel electrophoresis was performed in different steps during cloning, while a standard Tris-acetate-EDTA buffer, agarose powder of a molecular biology grade (Norgen Biotek Corp, Thorold, ON, Canada, cat. n. 28034) and the SYBR™ Green I Nucleic Acid Gel Stain (Thermo Fisher, Waltham, MA, USA, cat. n. S7563) intercalating dye were employed.

Two different percentages of agarose gel were used on the basis of the size of the DNA loaded, for example, 0.8% was used for the plasmid and digestion check and 1.8% was used for the insert check and colony PCR. Two different DNA ladders were used, the MassRuler DNA ladder (Thermo Fisher, Waltham, MA, USA, cat. n. SM0403) for 0.8% of gel and the GeneRuler 50 bp DNA ladder (Thermo Fisher, Waltham, MA, USA, cat. n. SM0373) for 1.8% of gel.

All the electrophoretic courses were conducted at 80V and imaged with Gel Doc EZ Gel Documentation System (Bio-Rad, Hercules, CA, USA), as previously reported [28].

### 2.10. Statistical Analysis

All the data were normally distributed and presented as mean ± SD The statistical differences between the groups were compared using a paired Student *t*-test. The statistical differences were considered significant when *p* < 0.05 (*), and they were considered highly significant when *p* < 0.01 (**) or when *p* < 0.001 (***). *p*: *p*-value.

## 3. Results

### 3.1. Characterization of Obtained K562 Clones

**Figure 1:**
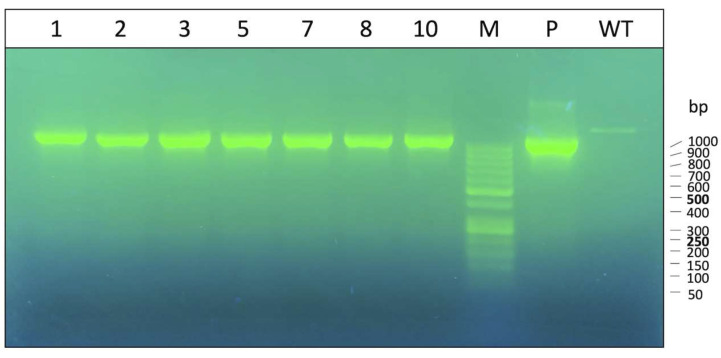
Check on Agarose gel of PCR products of obtained clones. The α-globin insert of the plasmid was amplified using primers on the plasmid sequence, ensuring that α-globin product came from cloning and not from K562 genome. We also amplified pcDNA3.1Hygro(+)-α-globin (P) as a positive control and K562 WT DNA as a negative control. Marker (M) graduation is displayed at the right of the gel image.

The construction of the pcDNA3.1Hygro(+)-α-globin plasmid is described in Figure S1 of Supplementary Materials. After performing transfection on this recombinant plasmid, K562 cells were selected using Hygromycin B at a 0.6 mg/mL final concentration, according to a previously plotted kill curve (Supplementary Figure S2). After checking the transfection on the cloned pool, cells were diluted and expanded from the single cells under the selection conditions. At the end of the process, seven stable clones were obtained and checked for the presence of pcDNA(α-glob)3.1/Hygro(+) plasmid (Figure 1).

**Figure 2:**
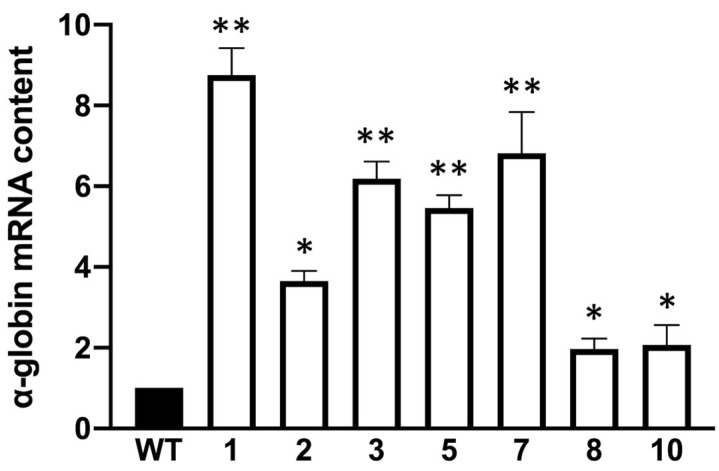
Summary showing α-globin mRNA content in obtained clones versus K562 wild-type cells (mean ± SD; N = 3). *p* < 0.05 (*), *p* < 0.01 (**).

All the tested clones were stably transfected and contained the prepared plasmid sequence inside their genome. The obtained K562 clones were further characterized according to α-globin mRNA levels using the RT-qPCR technique and three reference genes (RPL13a, GAPDH, β-actin). The representative data obtained by normalizing the clones with β-actin are displayed in Figure 2. The data obtained using RPL13a and GAPDH as reference sequences were very similar (Supplementary Figure S3).

**Figure 3:**
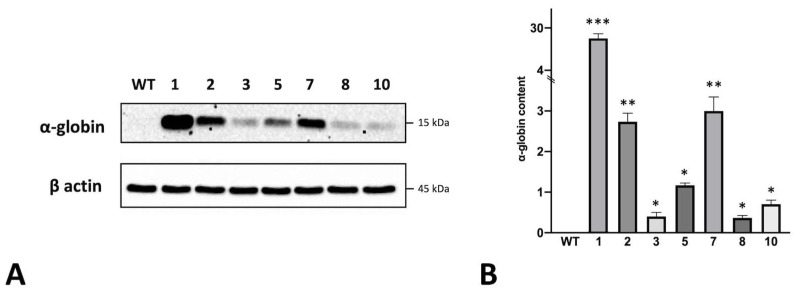
Representative Western blot experiment showing α-globin protein content in cellular lysates of K562 wild-type cells and obtained clones (**A**) and relative densitometry data set (**B**); data were normalized on β-actin (mean ± SD; N = 3). *p* < 0.05 (*), *p* < 0.01 (**), *p* < 0.001 (***).

All the tested clones showed a statistically significant increase in α-globin mRNA accumulation compared to the control WT-K562 cells (black histogram of Figure 2). For instance, clone 1 shows the highest fold increase (>8) together with clones 3, 5 and 7 (around 6-fold increase), while clones 2, 8 and 10 displayed a lower accumulation of α-globin mRNA. To support the data obtained with RT-qPCR, we performed Western blot analysis to ensure that the increase in the α-globin transcript in the tested clones corresponded to an increase in the encoded protein. It is well-known that the K562 cell line does not produce the α-globin protein, even if it is expressed in terms of transcripts, and probably among the other globin transcripts has minor success in competing for translation [21]. A Western blot was performed by loading 20 μg of protein extracts on 14% polyacrylamide gel, as explained in the methods section, which was transferred into the nitrocellulose membrane and incubated with a primary antibody against α-globin. A representative experiment is shown in Figure 3A; the uncropped version of this western blot is available in Supplementary Figure S4.

The experiment clearly showed that each of the obtained K562 cell clones was able to produce α-globin, although to different extents, while the Western blotting analysis of the original K562 cell line (WT) cells confirmed the above-mentioned literature findings [21], i.e., that K562 exhibits an α-thalassemia-like phenotype with the production of α-globin mRNA (Figure 2) but a lack of α-globin protein production. Clone 1, together with clones 7 and 5, produce the largest amount of α-globin, which is fully in agreement with the obtained RT-qPCR data. Surprisingly, clone 2 showed a good amount of α-globin protein, even if the transcript was not one of the highest; on the other hand, clone 3 showed a very low quantity of protein, despite the high amounts of α-globin transcripts. Clones 8 and 10 were confirmed as the minor α-globin protein producers, as expected from the RT-qPCR data.

### 3.2. Preliminary Studies on α-Globin Accumulation in Obtained Clones and Its Relationship with Autophagy

As a consequence of α-globin hyper-production, we investigated the autophagy process, which is primarily responsible for the degradation of most long-lived or aggregated proteins and cellular organelles.

**Figure 4:**
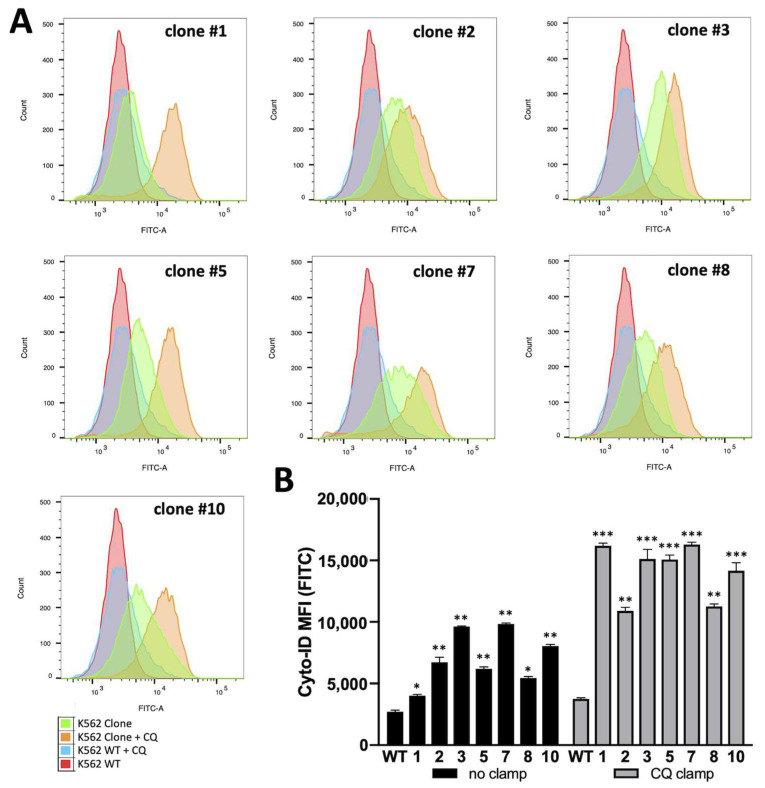
α-globin hyper-expression inducing autophagy in K562 cells, K562 wild-type cells (red curve) and tested clones (green curves) were clamped with chloroquine 40 μM for 48 h (blue and orange curves, respectively) to provide a clear shift in autophagy signal (Cyto-ID) (**A**). Autophagy levels in K562 cells and obtained clones hyper-expressing α-globin (mean ± SD; N = 3); basal autophagy levels are increased in clones (black histograms) and further increase blocking autophagosome degradation with chloroquine (grey histograms) (**B**). *p* < 0.05 (*), *p* < 0.01 (**), *p* < 0.001 (***).

Autophagy levels were measured in WT K562 cells and the obtained clones by flow cytometry, employing the Cyto-ID autophagy detection kit as mentioned in the methods section. The representative data obtained from the analysis are shown in Figure 4.

To better detect the autophagic flux, we used chloroquine to block autophagosome–lysosome fusion (clamp assay). In this way, the autophagosomes accumulated in the cells and the probe fluorescence would have increased much more if the autophagic process was triggered. The K562 WT cells (red curve), for example, exhibit a very low autophagic flux as chloroquine clamping (blue curve) did not produce any relevant shifts in the FITC signal (Cyto-ID probe). Conversely, all the obtained α-globin clones showed a higher basal signal (green curve) of the Cyto-ID probe, demonstrating a higher number of autophagic vacuoles. Furthermore, clamping with CQ dramatically raised the FITC signal in all the tested clones, confirming a high autophagic flux in all the α-globin-hyper-producing cells.

When the data from the FACS analysis (Figure 4) were correlated with the α-globin content (Figure 3), we found an appreciable correlation (r = 0.76), which was determined by Pearson correlation test (Supplementary Figure S5).

**Figure 5:**
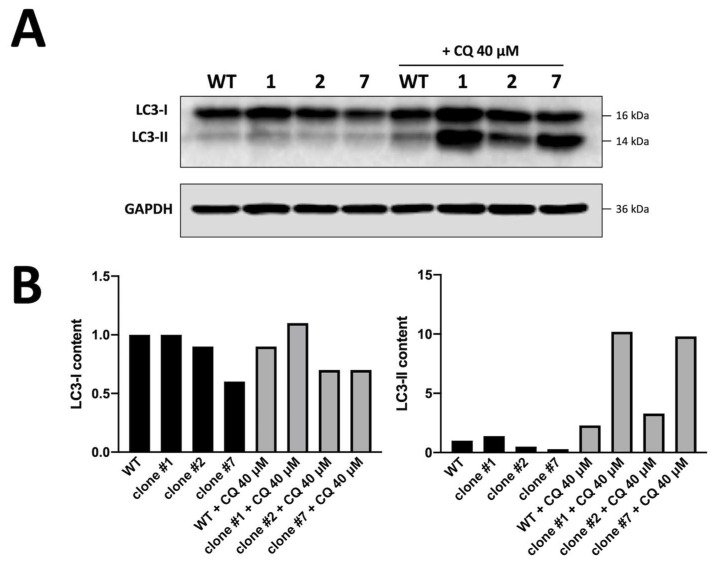
Western blotting experiment performed on clones 1, 2 and 7 (the producers of higher amounts of α-globin protein) and validating data obtained with FACS analysis; we employed chloroquine clamping for 48 h to better show autophagic flux (**A**). Summary showing LC3-I and LC3-II content in K562 WT cells vs. in clones 1, 2 and 7 when CQ was added for 48 h. LC3-II protein levels increased in obtained clones with respect to WT cells, confirming a higher number of autophagic vesicles, as founded by FACS analysis (**B**).

In addition, we confirmed the activation of the autophagy process by combining the FACS analysis with a different approach based on Western blotting assays. The results of the experiment are shown in Figure 5 and fully confirm the higher number of autophagic vesicles detected by the FACS analysis of K562 clones expressing the highest levels of α-globin compared to WT K562 cells. Uncropped version of this western blot is available in Supplementary Figure S6.

### 3.3. Biochemical and Molecular Characterization of Autophagy in K562 Cellular Clones Hyper-Expressing α-Globin

**Figure 6:**
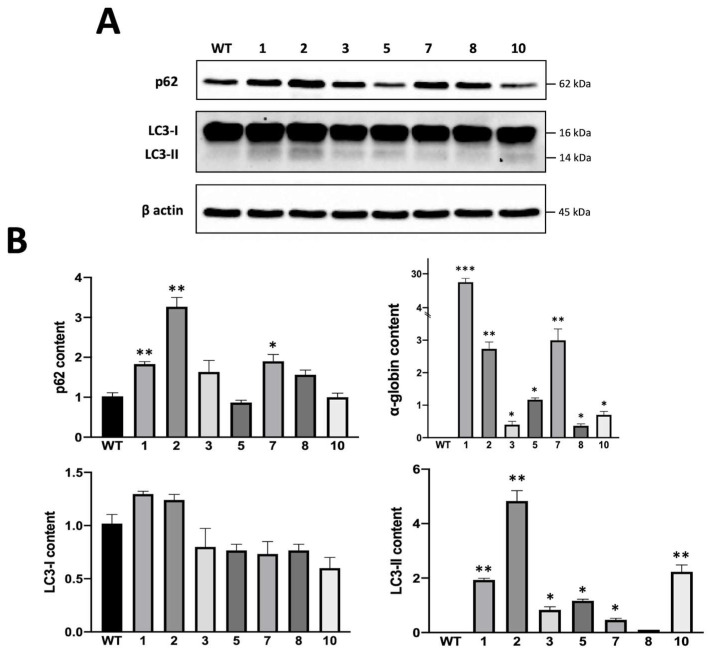
Representative Western blotting experiment showing p62 and LC3-I/II levels in K562 wild-type cells compared to those in obtained clones hyper expressing α-globin protein (**A**). Western blotting summary of p62 and LC3-I/II protein levels in K562 and obtained clones hyper-expressing α-globin. Soluble α-globin protein content is reported in the upper-right part of panel B to show the correlation between p62 and α-globin production in tested clones (mean ± SD; N = 3) (**B**). *p* < 0.05 (*), *p* < 0.01 (**), *p* < 0.001 (***).

Autophagy levels were also assessed by the Western blotting technique; we analyzed the cellular contents of the LC3 and p62 proteins. LC3 is a cytosolic protein (LC3-I) that during autophagy is conjugated to phosphatidylethanolamine to form LC3-phosphatidylethanolamine conjugate (LC3-II), which is recruited by the autophagosomal membranes; therefore, LC3-I downregulation and/or LC3-II upregulation is a signal of active autophagy in analyzed cells. The adaptor protein p62 (also called sequestosome1) is a protein that can bind damaged proteins or other ubiquitinated scaffolds and at the same time work as an adapter to bind the LC3 protein, therefore bringing to autophagosomes material to degrade; usually, p62 levels decrease during active phases of autophagy because the protein is actively degraded by autophagosomes, but the process is very quick and dynamic as a feedback mechanism is required to recharge the cells of this protein. A representative blot is shown in Figure 6; the uncropped version of this western blot is available in Supplementary Figure S4.

Each of the tested clones showed an increase in LC3-II protein compared to WT K562, indicating a higher number of autophagosomes. The accumulation of LC3-II protein was accompanied to a reduction in LC3-I content, as expected, in clones 3, 5, 7, 8 and 10, while in clones 1 and 2, it appears to have augmented together with LC3-II. Also, p62 levels were modulated in the tested clones; in general, p62 levels were higher in the obtained clones and especially in clones that showed higher α-globin protein levels (clones 1, 2 and 7). Only clones 5 and 10 showed a decrease in p62, probably due to the dynamic and active process of autophagolysosome degradation. A summary of the collected data is provided in Figure 6B. Interestingly, when the Western blotting analysis was performed using lysates from erythroid precursor cells isolated from β-thalassemia patients, higher levels of p62 were found in comparison with healthy donors and in association with high levels of free α-globin chains detected by HPLC.

**Figure 7:**
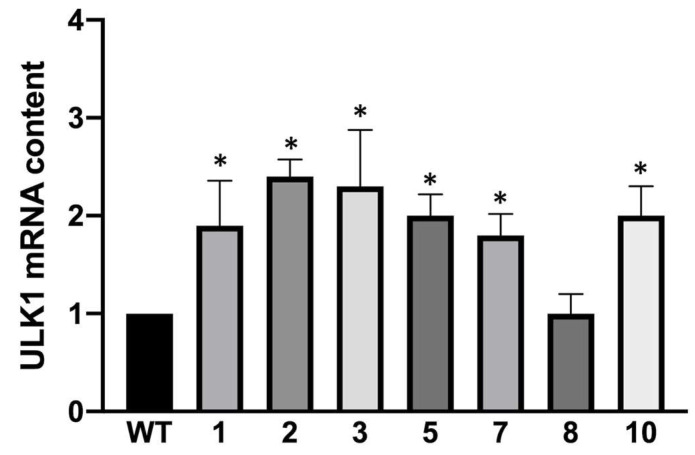
Summary representing the mean ULK1 mRNA content found in K562 wild-type cells and obtained clones (mean ± SD; N = 3); ULK1-dependent autophagy is linked to α-globin hyper-expression. *p* < 0.05 (*).

In support of these results, we have also tested the gene expression of ULK1, a serine/threonine kinase that is the most upstream component of the core autophagy machinery conserved from yeast to mammals. The results shown in Figure 7 support the findings obtained above as almost all the clones hyper-producing α-globin seemed to upregulate the ULK1 transcripts, and therefore, the increased autophagy levels encountered during the FACS experiments using the Cyto-ID autophagy detection kit are plausible.

### 3.4. Phenotypic Characterization of the K562 Cellular Clones Hyper-Expressing α-Globin: Stability of the Plasmid Integration and Tendency to Undergo Apoptosis

Finally, we obtained α-globin clones for two weeks without the Hygromycin B selection antibody to check if the plasmid integration was stable; moreover, we performed Annexin-V staining with a FACS apparatus in order to evaluate the cellular fitness of the clones hyper-producing α-globin protein.

**Figure 8:**
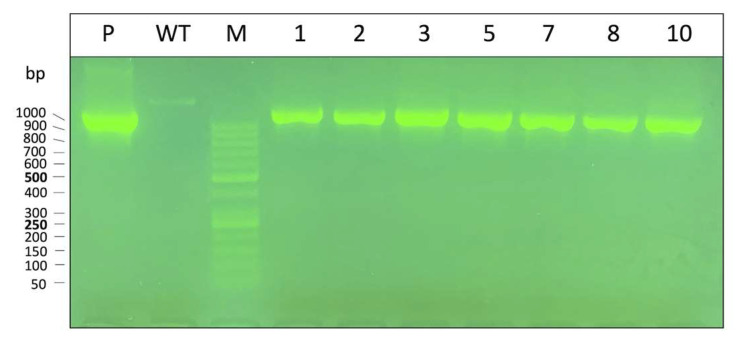
Check on agarose gel of PCR products of obtained clones cultured in absence of Hygromycin selection antibiotic; we amplified pcDNA3.1Hygro(+)-α-globin (P) as a positive control and K562 DNA as a negative control. Marker (M) graduation is displayed at the left of the gel image.

As depicted in Figure 8, in each one of the tested clones the α-globin plasmid DNA sequence (PCR product being 1100 bp using plasmid primers) was contained inside the extracted genomic DNA, demonstrating that the plasmid was integrated into the genome and not in an episomal form, since these clones did not lose the plasmid sequence when the selection antibiotic was removed.

**Figure 9:**
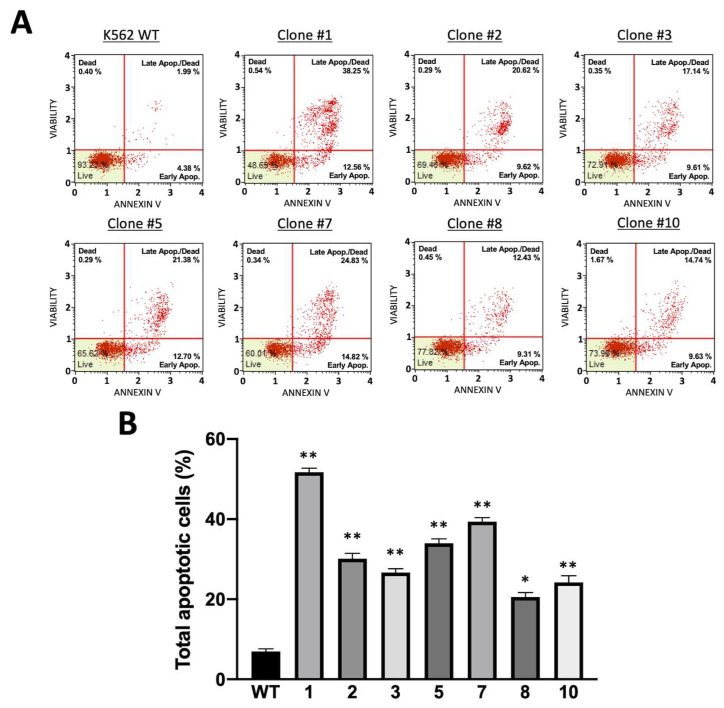
(**A**) Representative dot plot obtained from flow cytometric analysis of apoptosis. Each obtained clone was cultivated without Hygromycin selection antibiotics to evaluate apoptosis induced by α-globin hyper-production. (**B**) Summary of percentage increase in apoptotic cells in K562 clones hyper-expressing α-globin protein (mean ± SD; N = 3). *p* < 0.05 (*), *p* < 0.01 (**).

The Annexin-V staining of the obtained clones revealed the lower vitality of the cloned cells. These results were expected as α-globin accumulation produces toxic aggregates for cells and as the hemolysis of β-thalassemia-affected patients is principally due to this accumulation. A representative FACS dot plot is shown in Figure 9A.

Clone 1 had the highest percentage of cells in apoptosis, which was expected because this clone was the most powerful producer of the α-globin protein, together with clones 5 and 7. Clone 8, which typically produced lower amounts of α-globin protein, was also the clone with higher cellular vitality. The results are summarized in Figure 9B.

## 4. Discussion

One of the most relevant pathophysiological parameters of β-thalassemia is the deep imbalance between the production of α-globin and that of β-globin, which is associated with the high accumulation rate of free α-globin in erythroid cells. The α-globin/β-globin imbalance is responsible of the most important and most clinically relevant pathogenic events associated with β-thalassemia, which are hemolysis, ineffective erythropoiesis, chronic anemia/chronic hypoxia, compensatory hemopoietic expansion and iron overload, as pointed out in several studies/reviews [1,2,3,4,5,6] and as recently reviewed by Villalobos et al. [7].

In this context, the importance of reducing α-globin/β-globin imbalance targeting α-globin gene expression was recently fully demonstrated by Mettananda et al. [14], who applied the use of a CRISPR/Cas9 genome editing approach to delete a key α-globin gene enhancer, causing a reduction in α-globin expression and in the correction of the globin chain imbalance in CD34+ cells from β-thalassemia patients. The second study fully demonstrating the clinical relevance of interfering with the α-globin gene expression in β-thalassemia was recently published by Pavani et al. [15], who CRISPR/Cas9-edited the α-globin locus in human hematopoietic stem cells, achieving the correction of the pathological phenotype of β-thalassemia.

One strategy to reduce the accumulation of toxic α-globin in erythroid cells is the reactivation of γ-globin gene expression; in this way, the increased production of HbF can partially lead to a decrease in the content of the free α-globin chains [25,29]. Several inducers have been screened, characterized, and validated as having the ability to induce HbF, but this effect is not always related to a major decrease in free α-globin chains, as neo-synthesized γ-globin chains may be not enough to bind the totality of free α-chains present in these cells.

In this case, several cellular model systems for the screening of HbF inducers are available, such as the K562 erythroleukemia cell line [30] or the HUDEP-1 cell line derived from umbilical cord blood cells [31,32].

Although these cellular models are of great utility, they lack the typical hyper-accumulation of α-globin, which is the basis of the cellular damage responsible for hemolysis in β-thalassemia. Therefore, the lack of a cellular model for the study of α-globin accumulation led us to create K562 clones hyper expressing the gene encoding α-globin.

A pharmacological approach to decrease the excess α-globin chain accumulation, independent from α-globin gene targeting, has been proposed by Lechauve at al. [13], who demonstrated that a decline in excess free α-globin might occur with high efficiency following the drug-mediated activation of autophagy. The loss of the autophagy-activating Unc-51-like kinase 1 (ULK1) gene in β-thalassemic mice reduces the autophagic clearance of α-globin in red blood cell precursors and exacerbates disease phenotypes. Conversely, the rapamycin-mediated activation of autophagy was associated with ULK-1 induction and with a clinically relevant decrease in excess free α-globin in β-thalassemic mice ameliorating their pathological status.

In the present study, we produced and characterized K562 cellular clones that, unlike cells in the original K562 cell line, stably produce high levels of α-globin. During characterization, we found that these clones express some cellular, biochemical and molecular markers associated to autophagy. First, the obtained clones showed higher LC3-II levels compared to WT cells, indicating that autophagic vesicles are augmented in cells that hyper-express α-globin. This result was strongly confirmed during FACS experiments with the Cyto-ID autophagy assay: while the K562 WT cells exhibited very low autophagic flux when clamped with chloroquine, all the obtained clones showed not only high basal levels of autophagy but also a dramatic increase in the signal when clamped with chloroquine. Another result that supports the idea that autophagy is spontaneously triggered in obtained clones is that the ULK1 transcript was upregulated in each tested clone hyper-producing α-globin. We also evaluated the cellular vitality of clones that produced α-globin after two weeks of culturing in the absence of selection antibiotics and found that they showed a clear tendency to undergo apoptosis and a tendency to do so in a proportional manner according to the level of accumulation of α-globin.

Among the various clones obtained, clone 1 produced the highest amount of α-globin protein, mimicking the imbalance of this globin chain present in β-thalassemia erythroid cells. We believe that this clone may represent a good cellular model for subsequent works aimed at the reduction of free α-globin chains by reducing their synthesis and/or reducing their accumulation, i.e., targeting α-globin mRNA by antisense oligonucleotide or inducing protein degradation by proteasomal activation or via the autophagy process.

These clones might be proposed for studying, in detail, the relationship between autophagy and the decrease in excess free α-globin chains. This issue is highly relevant for defining and understanding the heterogeneous phenotypes in β-thalassemia patients [33]. In this context, excess free α-globin should be considered unstable and cytotoxic to erythroid cells (both precursors and mature cells). The proteasome is a key structure for the efficient degradation of polyubiquitinated unstable α-globin. If this activity is not sufficient, α-globin forms relatively insoluble aggregates acting as substrates for autophagy [34]. The study of the molecular cross-talk existing between these two pathways can take advantage of the use of the cellular model systems described here, and can be easily employed to further study the inhibition of proteasome activity resulting in the accumulation of unfolded proteins, the activation of stress pathways, and the consequent induction of autophagy and heat-shock/molecular chaperone responses.

As the first set of studies, we propose this new model system for the screening of pharmacological agents that are able to activate the full program of autophagy to reduce α-globin accumulation, but the model may be also suitable as a new therapeutical approach targeted at reducing the expression of α-globin genes to be eventually validated, extending the study to other available cell lines, such as HUDEP-1 and HUDEP-2 [31,32]. In particular, the HUDEP-2 cell line is of relevant interest, since, unlike K562 cells, its cells express predominantly adult β-globin and most closely resemble adult erythroid cells [32].

In addition, further studies are required to complete the analysis of all the molecules involved in the onset and control of the autophagic process; in fact, in the present study, autophagy was only partially analyzed (for a more complete list of the molecules involved in autophagy, see the Supplementary Table S4). Autophagy is a dynamic process requiring the careful monitoring of autophagic flux. Moreover, the involvement of ULK-1 is here presented only at the mRNA level. In order to fully understand the impact of ULK-1 and its modulation, in our experimental model systems, Western blotting analysis should be used in future studies in order to assess (a) the accumulation of the ULK-1 protein in relation to ULK-1 gene transcription and (b) the post-translational modifications (for instance phosphorylation) of ULK-1 or its targets affecting autophagy and mitophagy activation [35,36,37].

## Figures and Tables

**Table 1:** List of primers and probes with related sequences used to perform RT-qPCR analyses.

Primers and Probes	Sequences
forward primer α-globin	5′-CGACAAGACCAACGTCAAGG-3′
reverse primer α-globin	5′-GGTCTTGGTGGTGGGGAAG-3′
probe α-globin	5′-HEX-ACATCCTCTCCAGGGCCTCCG-BFQ-3′
forward primer ULK1	5′-CTACCTGGTTATGGAGTACTGC-3′
reverse primer ULK1	5′-GGAAGAGCCTGATGGTGTC-3′
probe ULK1	5′-FAM-CGACTACCT/ZEN/GCACGCCATGC-BFQ-3′
forward primer RPL13A	5′-GGCAATTTCTACAGAAACAAGTTG-3′
reverse primer RPL13A	5′-GTTTTGTGGGGCAGCATACC-3′
probe RPL13A	5′-HEX-CGCACGGTCCGCCAGAAGAT-BFQ-3′
forward primer ACTB	5′-ACAGAGCCTCGCCTTTG-3′
reverse primer ACTB	5′-ACGATGGAGGGGAAGACG-3′
probe ACTB	5′-Cy5-CCTTGCACATGCCGGAGCC-BRQ-3′
forward primer GAPDH	5′-ACATCGCTCAGACACCATG-3′
reverse primer GAPDH	5′-TGTAGTTGAGGTCAATGAAGGG-3′
probe GAPDH	5′-FAM-AAGGTCGGAGTCAACGGATTTGGTC-BFQ-3′

**Table 2:** Western blot primary and secondary antibodies employed for the detection of soluble α-globin chains and autophagy-related markers in cloned cells.

Target	Primary Antibody	Cat. n.	Secondary Antibody	Cat. n.
α-globin	Mouse anti-hemoglobin α (D-4) (Santa Cruz Biotechnology, Dallas, TX, USA)	sc-514378	Goat anti-mouse IgG HRP (Thermo Fisher, Waltham, MA, USA)	32430
p62	Rabbit anti-p62/SQSTM1 (Sigma-Aldrich, St. Louis, MO, USA)	P0067	Mouse anti-rabbit IgG HRP (Cell Signaling Technology, Danvers, MA, USA	7074
LC3	Rabbit anti-LC3B (Sigma-Aldrich, St. Louis, MO, USA)	L7543	Mouse anti-rabbit IgG HRP (Cell Signaling Technology, Danvers, MA, USA)	7074
β-actin	Rabbit anti-β-actin (Cell Signaling Technology, Danvers, MA, USA)	4967	Mouse anti-rabbit IgG HRP (Cell Signaling Technology, Danvers, MA, USA)	7074

## Data Availability

Materials and further information on the data will be freely available upon request from the corresponding authors.

## Supplementary Materials

The following supporting information can be downloaded at: https://www.mdpi.com/article/10.3390/genes14030556/s1. Supplementary methods: Detailed information about the cloning process. Figure S1: a summary of the adopted cloning strategy; Figure S2: the Hygromycin B kill curve; Figure S3: the α-globin gene expression data normalized with RPL13a and GAPDH; Figure S4: the uncut version of the Western blots presented in Figure 3 and Figure 6; Figure S5: a correlation analysis taking in consideration the data obtained by the FACS analysis on autophagy and the α-globin content of the obtained clones; Figure S6: the uncut version of the Western blot presented in Figure 5; Table S4: a summary of the key molecules involved in autophagy [38,39,40,41,42].
