# Supplementary material for: Production and Characterization of K562 Cellular Clones Hyper-Expressing the Gene Encoding α-Globin: Preliminary Analysis of Biomarkers Associated with Autophagy
Source: Genes (Basel). 2023 Feb 23;14(3):556. doi: 10.3390/genes14030556 (PMC10048432; doi:10.3390/genes14030556)
Supplement: Supplementary file 1 [file genes-14-00556-s001.zip › genes-2149620-supplementary.pdf]

Article

Production and characterization of K562 cellular clones hyper-expressing the gene encoding α-globin: preliminary analysis of biomarkers associated with autophagy

Matteo Zurlo[1], Jessica Gasparello[1], Lucia Carmela Cosenza[1], Giulia Breveglieri[1], Chiara Papi[1], Cristina Zuccato[1,2], Roberto Gambari[1,2] and Alessia Finotti[1,2]*

[1] Department of Life Sciences and Biotechnology, Section of Biochemistry and Molecular Biology, sity of Ferrara, 44121 Ferrara, Italy

[2] Center 'Chiara Gemmo and Elio Zago' for the Research on Thalassemia, University of Ferrara, 44121 Ferrara, Italy

* Correspondence: alessia.finotti@unife.it (A.F.) Tel.: +39-0532-974510

SUPPLEMENTARY MATERIAL

Supplementary methods

1.1. Obtaining ultracompetent JM-109 bacteria

Chemically obtained competent bacteria were prepared starting from culturing Escherichia coli JM-109 (Promega, Milan, Italy, cat. n. P9751) non-competent bacteria overnight in handmade Luria-Bertani LB medium at 37°C in mild agitation in a culture tube. The day after, 2 ml of bacteria were placed in handmade Psi Broth medium pH7.6 and amplified at 37°C in mild agitation for 2 hours. The bacteria growth was periodically measured with Spectrophotometer in order to start the procedure with bacteria in full logarithmic phase of growth. Then, the bacterial suspension was aliquoted in four 50 ml falcon tubes and placed in ice 15 minutes, after incubation tubes were centrifuged 20 minutes at 3000 rpm, 4°C. Supernatants were discarded and bacterial pellet were resuspended using 20 ml of pre-chilled Tfb1 buffer pH5.8 (100 mM RbCl, 10 mM CaCl2, 50 mM MnCl, 20 mM CH3CO2K, 15% Glycerol) and incubated 15 minutes in ice. After 20 minutes of centrifugation at 3000 rpm 4°C, supernatants were discarded, and bacterial pellet was resuspended with 2 ml of ice-cold Tfb2 buffer pH6.5 (10mM MOPS, 75 mM CaCl2, 10 mM RbCl, 15% Glycerol); finally, ultracompetent bacteria obtained were aliquoted in several 150 µl aliquots and immediately put in dry ice and stocked at -80°C until use.

1.2. Plasmid digestion and purification

pcDNA3.1/Hygro(+) plasmid was prepared by digestion with NheI e XbaI restriction enzymes (Fermentas by Thermo Fisher, Waltham, MA, USA, cat. n. ER0975 and ER0681). The reaction was performed in 20 µl using 2 µg of pcDNA3.1/Hygro(+) plasmid, 2 µl of Buffer Tango 10x, 1 µL of NheI (10 U/µl), 1 µL of XbaI (10 U/µL), and nuclease-free water. Enzymatic digestion was carried out at 37°C for 1 h and followed by inactivation of the enzymes at 65°C for 20 minutes. Reactions performed were checked by 0,8% agarose gel electrophoresis.

The digested products were isolated from the gel using the Expin Gel SV gel extraction kit (GeneAll Biotechnology, Seoul, South-Korea, cat.n. 102-150), collected together and purified using microclean reagent (Clent Lifescience, Amblecote, UK, cat. n. 2MCL-1).

1.3. Genomic DNA isolation from whole blood

Genomic DNA isolation from whole blood was performed with GRS Genomic DNA kit (GRiSP, Porto, Portugal, cat. n. GK06.0100) following manufacturers protocol; extracted genomic DNA was furthermore purified using microclean reagent (Clent Lifescience, Amblecote, UK).

1.4. High fidelity PCR, digestion, and purification for α-globin insert production

Insert was obtained thanks to High Fidelity Polymerase enzyme (KAPA HiFi HotStart readymix, Roche diagnostics, Basel, Switzerland, cat. n. KK2601) and a couple of primers specifically designed to provide a successful sticky ligation with the plasmid exploiting NheI and XbaI restriction sites as shown in Supplementary Tab. S1 (IDT, Castenaso, Italy). The final reaction mix contained 80 ng of blood-extracted purified genomic DNA, 100 ng of NheI Fw primer and 100 ng of XbaI Rv primer, KAPA HF mix 1x and nuclease-free water. The amplification protocol was the following: 95°C for 5 m, 98°C 20s-62°C 15s-72°C 30s repeated for 30 times, 72°C for 5 m. The amplification product was verified by 1,8% agarose gel electrophoresis and the rest of the amplified material was co-digested with NheI and XbaI and purified with microclean reagent (Clent Lifescience, Amblecote, UK) as explained above.

Table S1. Sequences of primers employed for α-globin gene insert obtaining by Hi Fidelity PCR, restriction enzyme sites are underlined.

Primers	Sequences
primer forward NheI	5′ - GGTGGT<u>GCTAGC</u>GTCCCCACAGACTCAGAGAGAA - 3′
primer reverse XbaI	5′ - GCAGCA<u>TCTAGA</u>GCCGCCCACTCAGACTTTATT - 3′

1.5. Ligation reaction and transformation

In order to insert cloned α-globin sequence into pcDNA3.1/Hygro(+) vector, ligation was performed using T4 DNA ligase (Thermo Fisher, Waltham, MA, USA, cat. n. 15224090). The 20 µl ligation reaction mixture contained: 50 ng of digested and purified pcDNA3.1(+), 25 ng of digested and purified α-globin insert, 2 µl of T4 DNA ligase buffer, 1 U of T4 DNA ligase (Fermentas by Thermo Fisher, Waltham, MA, USA) and nuclease-free water. The reaction was carried out for 3 h at 22°C, overnight at 16°C, 8 h at 4°C and stopped by heating for 10 minutes at 65°C before starting the transformation procedure.

Transformation procedure starts slowly defrosting a stock aliquot of JM109 ultracompetent bacteria in ice, then 15 µl of ligation reaction were rapidly pipetted into ultracompetent bacteria and placed in ice for 30 minutes. After incubation, the sample was subjected to thermal shock by putting it in water bath at 42°C for 45 seconds and then immediately 2 minutes in ice; the bacteria were harvested and diluted in LB medium and rapidly placed in incubation in a culture tube at 37°C in moderate agitation for 1 h. Expanded bacteria were finally plated in a LB-agar plate containing Ampicillin 100 µg/ml (Sigma-Aldrich, St Louis, MO, USA, cat. n. A9393) and incubated at 37°C, 5% CO2 overnight.

The next day, plates presenting colonies following the transformation process were packed with parafilm and stored at 4°C until further characterization analysis.

1.6. Colony PCR

Colonies were picked up from ligation plates and dissolved in 20 ul of nuclease-free water, 5 µl of each colony solution was used as template for PCR colony reaction containing the following mix: 100 ng of CMV Fw primer, 100 ng of α-globin Rv primer, 1x Wonder Taq reaction Buffer, 5 U of Wonder Taq polymerase (Euroclone, Pero, Italy, cat. n. EME020001) and nuclease free water. The amplification protocol followed was the following: 98°C 5 m, 95°C 30s-62°C 30s-72°C 30s repeated for 30 cycles, 72°C 10'.

The strategy of employing one primer on the insert sequence (α-globin) and one primer on the plasmid sequence (CMV) during PCR (Supplementary Tab. S2) allows to discriminate only colonies that come from successfully ligation, PCR products were checked by 1,8% agarose gel electrophoresis.

Table S2. Sequences of primers employed for colony PCR reaction.

Primers	Sequences
primer forward CMV	5′ - GTCCCCACAGACTCAGAGAGAA - 3′
primer reverse α-globin	5′ - GCCGCCCACTCAGACTTTATT - 3′

1.7. Plasmid purification from bacteria and insert sequencing

Bacteria derived from positive colonies were amplified overnight in LB medium added of Ampicillin and the day after were extracted using QIAGEN Plasmid Midi kit (QIAGEN, Hilden, Germany, cat. n. 28706) following manufacturer instruction. The insert of the obtained plasmid was checked by agarose gel electrophoresis and sequenced in order to ensure the quality of the work. PCR reaction for sequencing was carried out using the Ex Taq polymerase (TaKaRa Bio Inc., Kusatsu, Japan, cat. n. RR01CM), the final reaction mixture contained: 1 μg of purified plasmidic DNA, 10 μM CMV Fw primer, 10 μM BGH Rv primer, dNTPs mix (2,5mM each), Ex Taq buffer 1x, 1,25 U of Ex Taq polymerase and nuclease-free water (see Supplementary Tab.3). The amplification protocol employed was the following: 94°C 2m, 94°C 30s-58°C 30s-72°C 1m30s repeated for 30 cycles, 72°C 10m. Products of amplification were then purified with microclean reagent (Clent Lifescience, Amblecote, UK) and resuspended in sterile nuclease-free water.
Sequencing reactions were carried out according to Sanger's method with the same primers employed for PCR amplification (Supplementary Tab. S3). The reactions were performed in a final volume of 20 μL containing 100 ng of purified PCR template, 25 ng of sequencing primer and 8 μL of Terminator Ready Reaction Mix of ABI PRISM® BigDye™ Terminator Cycle Sequencing Ready Reaction Kit (Thermo Fisher, Waltham, MA, USA, cat. n. 4337456). A total of 45 amplification cycles were performed as follows: denaturation, 96 °C, 10 s; annealing, 58 °C, 5 s; elongation, 60 °C, 4 min. After purification of the reaction products using Sephadex™ G-50 Superfine (Sigma-Aldrich, St Louis, MO, USA, cat. n. G5050), sequencing was finally performed by BMR Genomics (Padua, Italy); the obtained sequencing data were analyzed with SnapGene v6.0 software (Dotmatics, Boston, MA, USA).

Table S3. Sequences of primers employed for α-globin gene insert sequencing reaction.

Primers	Sequences
primer forward CMV	5′ - TAGGCGTGTACGGTCGGA - 3′
primer reverse BGH	5′ - TTAGGAAAGGACAGTGGGAGTGG - 3′

1.8. K562 Hygromycin kill curve, cells transfection and selection

To ensure selection of transfected cells, the optimal concentration of selection antibiotic must be found by performing a kill curve. For this purpose, K562 cells were seeded in a 24 well plate ($1x10^6$ cells/ml) in 0,5 ml of standard RPMI-1640 medium supplemented with 10% FBS and treated with increasing concentrations of Hygromycin B selection antibiotic (MedChemExpress, Monmouth Junction, NJ, USA, cat. n. HY-B0490). Cells were cultivated for ten days and then the cellular viability was determined by cell count and Trypan Blu; the minimum quantity that kills all the cells in ten days was 0,6 mg/ml in our experimental conditions (Supplementary Fig. S2).

K562 cells were seeded in a 24-well plate ($200x10^3$ cells/ml) in 0,5 ml of standard RPMI-1640 medium supplemented with 10% FBS and transfected using Lipofectamine LTX reagent (Invitrogen by Thermo Fisher (Waltham, MA, USA, cat. n. A12621). Briefly, 500 ng of purified preparade plasmid was incubated with 0,5 µl of PLUS reagent in 50 µl of Opti-MEM medium (Gibco by Thermo Fisher, Waltham, MA, USA, cat. n. 31985070) for 10 m at room temperature, then 2 µl of Lipofectamine LTX were added and the mix incubated other 30 m at room temperature. Finally, cells were treated with liposome-plasmid mix and incubated for 48h in humidified atmosphere at 37°C with 5% CO2. After this incubation period, cells were washed and resuspended in fresh RPMI medium containing 20% FBS and added with Hygromycin 0,6 mg/ml to ensure selection of transfected cells. After two weeks of culturing, cells were washed, resuspended, and selected by limiting dilutions; this was achieved by diluting cells many times in RPMI medium containing 20% FBS and Hygromycin 0,6 mg/ml and plating in several 96 well plates at a final volume of 100 µl per well. Plated cells were checked under the microscope to detect every well containing a single cell and noted. After about ten days of expansion in selection medium, cells expanded from single cells noted were tested for plasmid presence in isolated genomic DNA by PCR and agarose gel electrophoresis; positive clones were amplified and stocked in liquid nitrogen and then further characterized by RT-qPCR and Western blotting techniques.

Supplementary Figures

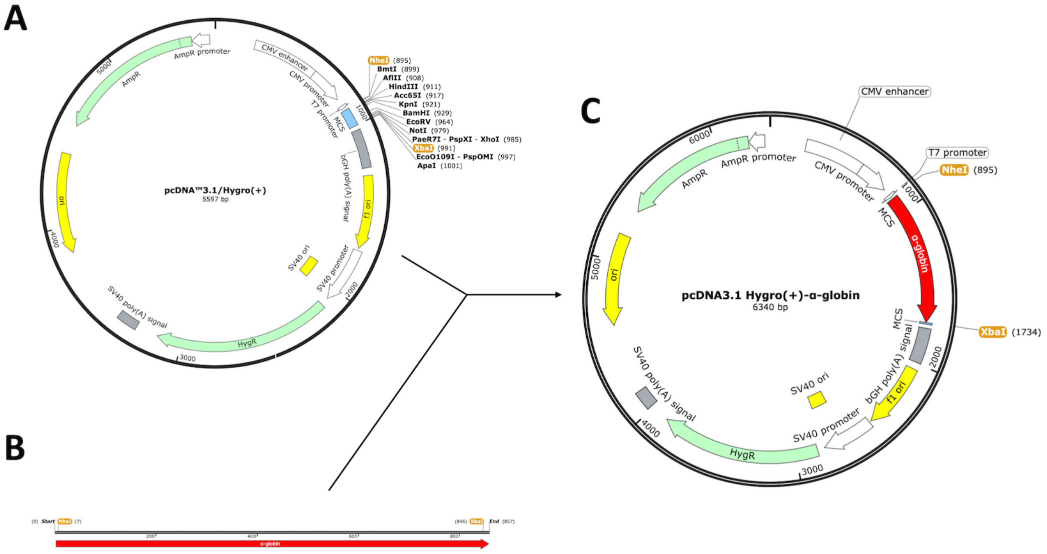

Figure S1. Summary of the adopted cloning strategy: pcDNA™3.1/Hygro(+) was digested with NheI and XbaI restriction enzymes (A) while α-globin gene sequence was obtained by High-Fidelity PCR reaction employing specific primers designed to introduce NheI and XbaI restriction sites in 5′ and 3′ respectively (B). Ligation reaction of the α-globin gene insert and the linearized vector was performed as described in detail in supplementary methods employing a T4 DNA Ligase (C).

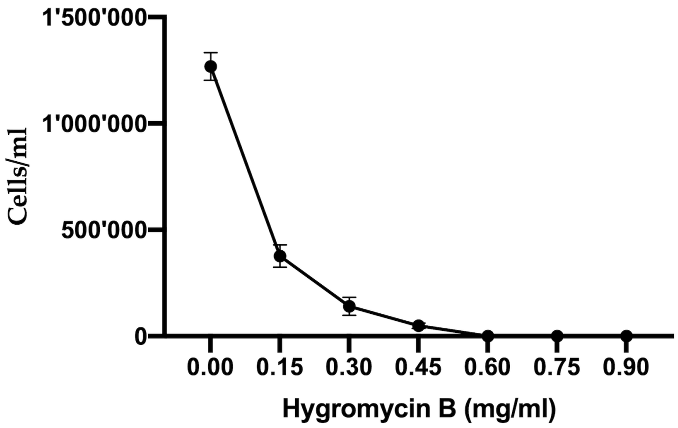

Figure S2. Hygromycin B Killing curve: the right concentration of selection antibiotic was tested in K562 WT and the minimum concentration able to kill all plated cells in 7 days was 0.6 mg/ml (mean ± SD; N = 3).

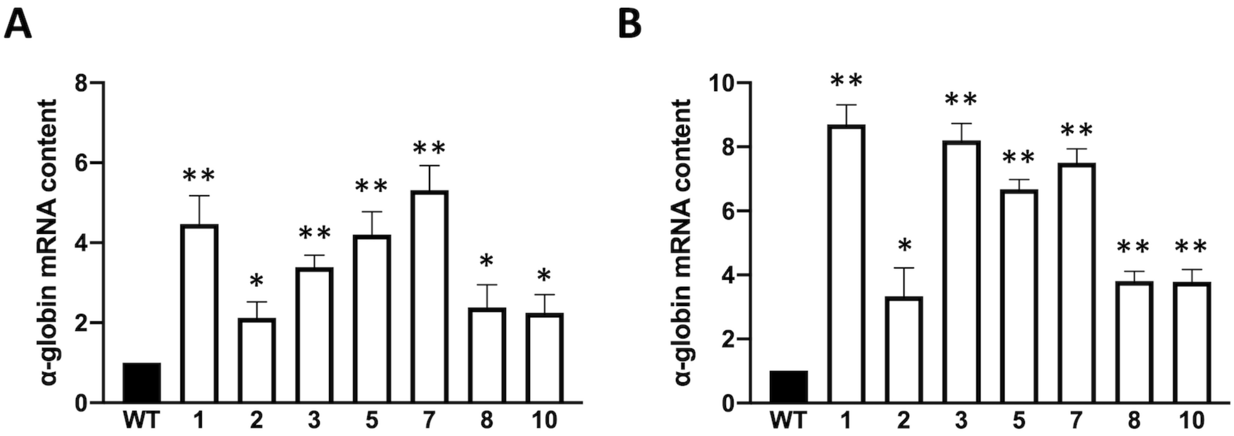

Figure S3. α-globin gene expression data normalized with RPL13A and GAPDH (mean ± SD; N = 3).

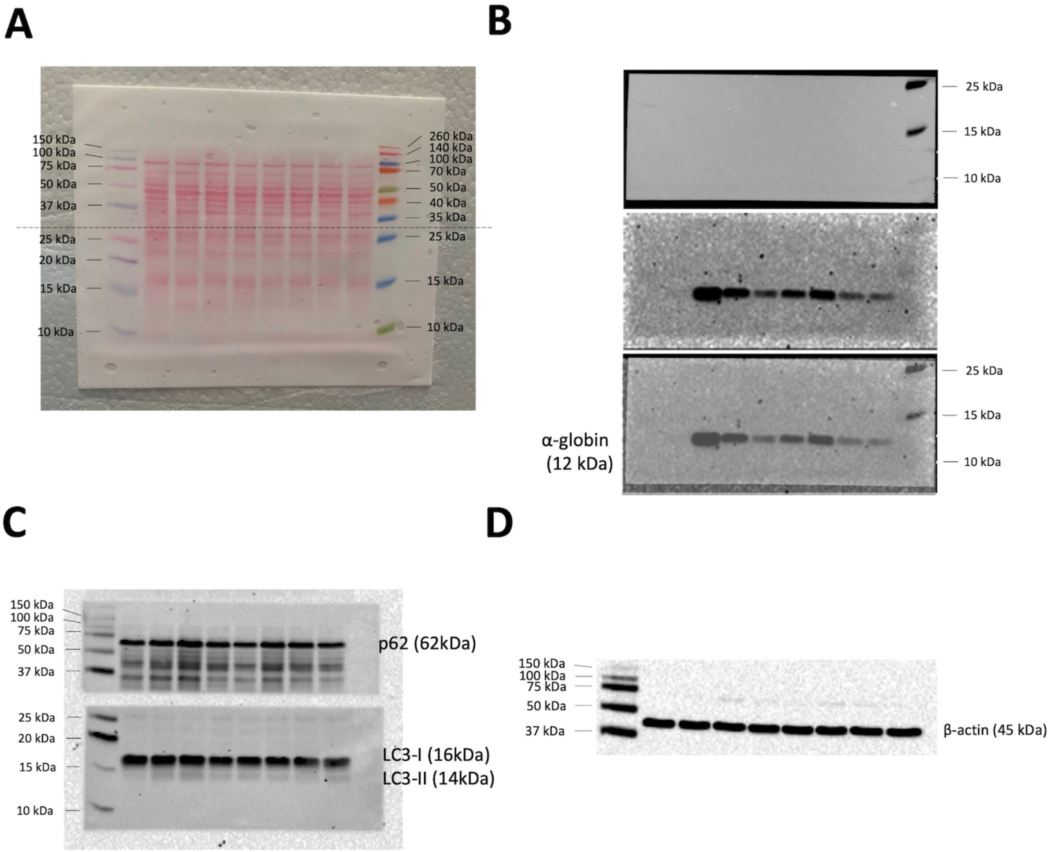

Figure S4. Uncut version of presented Western blots in Fig.3 and Fig.6. A. Ponceau S staining of the nitrocellulose membrane after the electrotransfer, on the left we indicated molecular weight represented by Bio-Rad Precision Plus Protein WesternC Standard (Bio-Rad, Hercules, CA, USA, cat. n. 1610376), while on the right we did the same for Spectra pre-stained ladder (Thermo Fisher, Waltham, MA, USA, cat. n. 26634); the dotted line represents the exact point where we cut the membrane to proceed with different staining in parallel. B. In order from top to bottom: pictures showing spectra marker in the lower piece of membrane, pictures showing α-globin development, merge of the two pictures showing the expected molecular weight for α-globin band. C. Development of p62 (higher piece of membrane) and LC3 (lower piece of membrane), in this case, Bio-Rad marker clearly show the exact molecular weight expected for the two proteins of interest. D. Development of β-actin (higher piece of membrane), even in this case Bio-Rad marker clearly shows the exact molecular weight expected for β-actin.

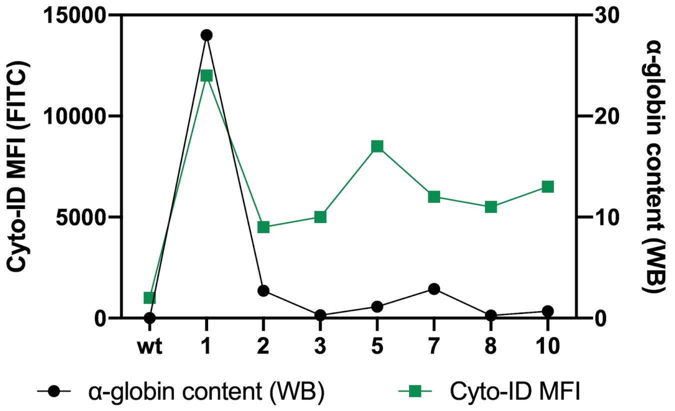

Figure S5. Correlation analysis taking in consideration data obtained by FACS analysis on autophagy (Figure 4) and the α-globin content (Figure 3) of obtained clones (r=0.76, p=0.028 determined by Pearson correlation test).

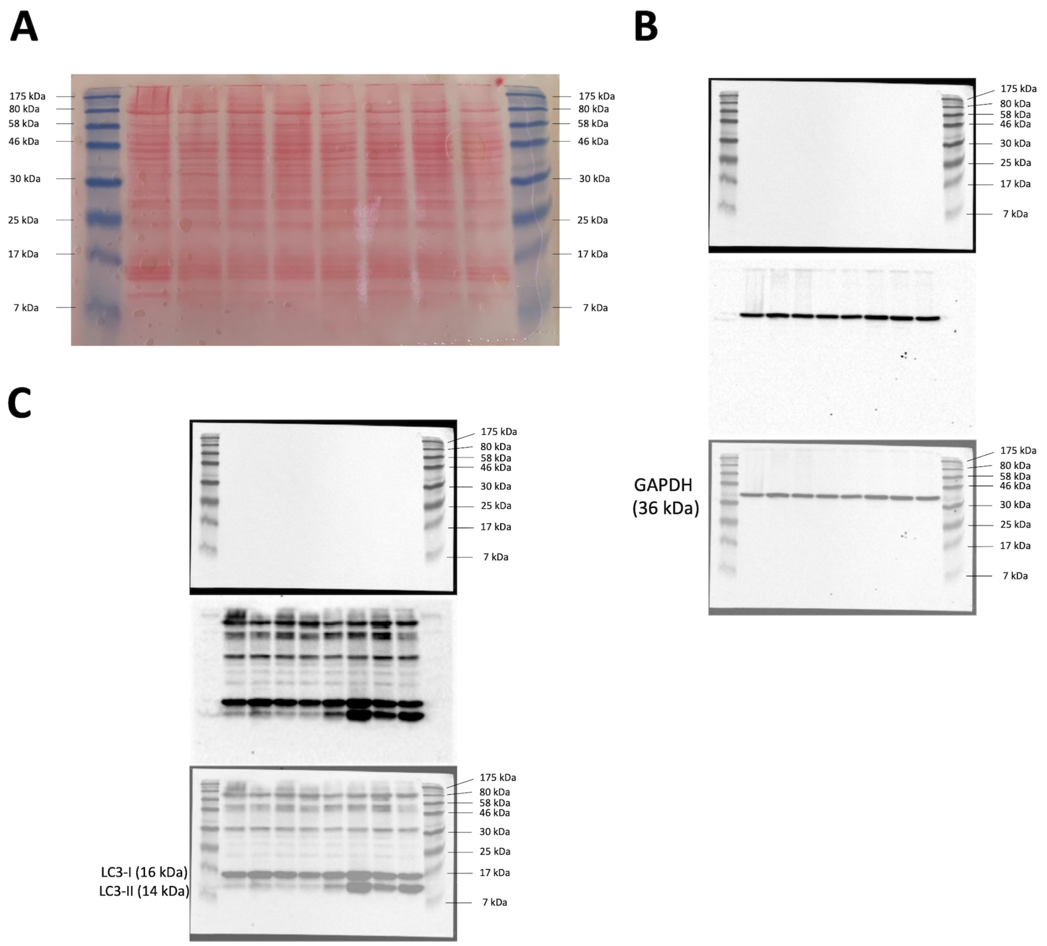

Figure S6. Uncut version of presented Western blots in Fig.5. A. Ponceau S staining of the nitrocellulose membrane after the electrotransfer, on the left we indicated molecular weight represented by the pre-stained protein marker (New England Biolabs, Ipswich, MA, USA, cat. n. P7708S) B. In order from top to bottom: picture showing NEB marker, picture showing GAPDH development, merge of the two pictures showing the expected molecular weight for GAPDH band. C. In order from top to bottom: picture showing NEB marker, picture showing LC3 development, merge of the two pictures showing the expected molecular weight for LC3 bands.

Supplementary Tables

Table S4. Summary of key players molecules involved in autophagy.

Target	*Function*	*Reference*
**mammalian target of Rapamycin (mTOR)**	Inhibition of mTOR signaling is associated with autophagy induction	Kim YC, Guan KL. mTOR: a pharmacologic target for autophagy regulation. J Clin Invest. 2015;125(1):25-32. doi:10.1172/JCI73939
**unc-51 like autophagy activating kinase 1 (ULK1)**	ULK1 is a key player in the autophagy initiation complex together with Fip200 and Atg13, moreover unphosphorylated ULK1 drives the fusion of autophagosome to lysosomes	Wang C, Wang H, Zhang D, et al. Phosphorylation of ULK1 affects autophagosome fusion and links chaperone-mediated autophagy to macroautophagy. Nat Commun. 2018;9(1):3492. Published 2018 Aug 28. doi:10.1038/s41467-018-05449-1
**beclin-1 (BECN1)**	During initial steps in the assembly of autophagosomes, the recruitment and activation of PI3K-III consisting of BECN1, VPS34, VPS15, and ATG14 is required to form the isolation membrane of the autophagosomes.	Sun Q, Fan W, Zhong Q. Regulation of Beclin 1 in autophagy. Autophagy. 2009;5(5):713-716. doi:10.4161/auto.5.5.8524
**p62 (SQSTM1)**	p62 is a cargo protein destined to be degraded by autophagy, including ubiquitinated protein aggregates destined for proteasome degradation. The p62 protein can bind also to LC3, thereby targeting the autophagosome and facilitating clearance of ubiquitinated proteins.	Liu WJ, Ye L, Huang WF, et al. p62 links the autophagy pathway and the ubiqutin-proteasome system upon ubiquitinated protein degradation. Cell Mol Biol Lett. 2016;21:29. Published 2016 Dec 13. doi:10.1186/s11658-016-0031-z
**ATG3, ATG4, ATG5, ATG7, ATG12, ATG16L Rab1, Rab5, Rab7, Rab9A, Rab11, Rab23, Rab32, and Rab33B**	All these numerous ATG proteins are involved in the machinery of lipidation of LC3-I protein to form LC3-II. Also, various Rab proteins participate during autophagosome formation.	Tanida I, Ueno T, Kominami E. LC3 and Autophagy. Methods Mol Biol. 2008;445:77-88. doi:10.1007/978-1-59745-157-4_4
**LC3-A/B**	LC3-I is conjugated to phosphatidylethanolamine to form LC3-phosphatidylethanolamine conjugate (LC3-II), which is recruited to autophagosomal membranes. This complex acts as link for p62 and proteins to be degraded.	Tanida I, Ueno T, Kominami E. LC3 and Autophagy. Methods Mol Biol. 2008;445:77-88. doi:10.1007/978-1-59745-157-4_4
**Rab9**	Rab9 is required in non-canonical autophagy and replace the role of LC3 system.	Saito T, Nah J, Oka SI, et al. An alternative mitophagy pathway mediated by Rab9 protects the heart against ischemia. J Clin Invest. 2019;129(2):802-819. doi:10.1172/JCI122035
